# Continuous spatial field confocal thermometry using lanthanide doped tellurite glass

**DOI:** 10.1038/s41598-024-65529-9

**Published:** 2024-07-02

**Authors:** Daniel Stavrevski, E. P. Schartner, Q. Sun, I. S. Maksymov, R. A. McLaughlin, H. Ebendorff-Heidepriem, A. D. Greentree

**Affiliations:** 1 Australian Research Council Centre of Excellence for Nanoscale BioPhotonics, Melbourne, Australia; 2 School of Science, RMIT University, Melbourne, VIC 3000 Australia; 3 School of Physics, Chemistry and Earth Sciences, The University of Adelaide, Adelaide, SA 5005 Australia; 4 Institute for Photonics and Advanced Sensing, The University of Adelaide, Adelaide, SA 5005 Australia; 5 Artificial Intelligence and Cyber Futures Institute, Charles Sturt University, Bathurst, NSW 2795 Australia; 6 Adelaide Medical School, The University of Adelaide, Adelaide, SA 5005 Australia

**Keywords:** Imaging and sensing, Micro-optics, Fluorescence spectroscopy

## Abstract

Distinguishing between microscopic variances in temperature in both space and time with high precision can open up new opportunities in optical sensing. In this paper, we present a novel approach to optically measure temperature from the fluorescence of erbium:ytterbium doped tellurite glass, with fast temporal resolution at micron-scale localisation over an area with sub millimetre spatial dimensions. This confocal-based approach provides a micron-scale image of temperature variations over a 200 $\upmu$m $\times$ 200 $\upmu$m field of view at sub-1 second time intervals. We test our sensing platform by monitoring the real-time evaporation of a water droplet over a wide field of view and track it’s evaporative cooling effect on the glass where we report a net temperature change of 6.97 K ± 0.03 K. This result showcases a confocal approach to thermometry to provide high temporal and spatial resolution over a microscopic field of view with the goal of providing real-time measures of temperature on the micro-scale.

## Introduction

Biological, chemical and industrial processes can be monitored by measuring the temperature change accompanied by the processes. For example, thermal imaging cameras have been used to monitor electrochemical reactions in fuel cells^1^. However, for most cases of infrared thermometry, the point to point resolution is in the millimeter scale, which is insufficient for monitoring the processes that occur on the micron-scale such as the formation of an initial ‘hot spot’ in semiconductor devices, which can cause thermal runaway^2,3^ that can ruin an entire device. Another example are self-sustaining chemical reactions that can be initiated from the combination of two molecules, with corresponding local temperature variation^4^. In biological systems, temperature variation may be indicative of disease progression, metabolism^5^ and inflammation^6,7^. The monitoring of processes at the micron-scale requires fast temperature measurement with high spatial resolution. Sensing approaches based on temperature dependent fluorescent transitions emitting light in the visible spectrum are particularly suited for such measurement requirements as visible fluorescence allows using widely available optical microscopy techniques such as confocal microscopy to achieve high spatial resolution.

The temperature, temporal and spatial resolution of fluorescence based sensing approaches depends on the read-out system and properties of the temperature sensitive fluorescence material, while the long-term stability depends also on the chemical and physical stability of the fluorescent compound. Organic molecules based fluorescent compounds such as such as Mito thermos yellow and Mito-RTP^8,9^ are able to bind to the mitochondria of a cell. This phenomenon combined with microscopy based read-out systems has allowed highly localised and fast monitoring of thermal processes related to a cell’s metabolism with a resolution of 0.1 K. An advantage to this approach is that measurements are taken directly from the region of interest giving real-time information on a cells temperature^10^ at small discrete points in space. A potential confound of these fluorophores is their low chemical stability, requiring well controlled environment. For example, the Green fluorescent protein requires the pH to be in a narrow range to function as fluorescence based sensor^11^. Inorganic nanoparticles such as fluorescent upconversion nanocrystals^12^, quantum dots^13–16^ and nitrogen vacancy containing diamond nanoparticles^17^ are viable alternatives as they offer higher chemical robustness, while their nanoscale size ensures highly localised temperature measurement as for the organic fluorescent molecules based sensing approaches. This has been demonstrated for point-based temperature measurements in various chemical and biological systems with sub-micron resolution^17^. The temperature, temporal and spatial resolution of fluorescence based sensing approaches depends on the read-out system and properties of the material for the fluorescence, while the long-term stability depends also on the chemical and physical stability of the fluorescent compound.

Of the various fluorescence species available for temperature sensing, rare earth ions are particularly suited as they demonstrate fluorescence with high brightness and long-term stability if the ions are incorporated into chemically robust materials such as inorganic crystals or glasses. Recently, there has been a large amount of work to develop rare earth doped glasses and upconversion nanocrystals with high temperature sensitivity^18–21^. In particular, erbium ytterbium (Er-Yb) doped glasses and nanocrystals have been widely used since the erbium ions have two green fluorescence bands that are thermally coupled, making the green fluorescence ratio a reliable temperature sensitive parameter. The co-doping with ytterbium allows use of the upconversion process to excite the green fluorescence with infrared light at 980 nm, which prevents undesired autofluorescence from the sample to be measured. For localised temperature measurement, the temperature sensitive Er-Yb doped materials were used as glass micro-particles, glass coating on an optical fibre tip or upconversion nanocrystals in different sensing configurations^22–26^.

An example of point temperature sensing is the use of an Er-Yb doped tellurite glass as a thin coating on the end of a conventional optical fibre that was implanted into the brain of a rat^22^ or inserted into brain tissue^23^. This fibre tip based configuration allowed in-vivo point sensing of intracranial temperature of a moving rat and combining imaging and sensing of different regions in tissue in the temperature range of 25–45 $^{\circ }$C with high sensitivity ($\approx 10^{-3}$ K$^{-1}$ ).

For localised temperature sensing of specific regions in a sample, Er-Yb doped upconversion nanocrystals were widely used^24–26^. The nanocrystals were placed in close proximity of or within the sample that was imaged using scanning confocal microscopy, combined with spectral measurement of the green fluorescence of the erbium ions in the nanocrystals. For example, isolated nanocrystals scattered in close proximity of a single silver nanowire were used to monitor the temperature of the nanowire, whereby the scanning confocal microscope system determined the spatial resolution to be in the order of 100 nm with an uncertainty in temperature measurements of $\pm 5$ $^{\circ }$C^24^. Other examples involved the use of nanocrystals that were engulfed by cells via endocytosis to monitor the temperature of cell organelles in the range of 5–60 $^{\circ }$C with sensitivity of 10$^{-3}$ K$^{-1}$^25,26^. Each of these demonstrations of thermometry, using either a glass coating on a fibre tip or upconversion nanocrystals, relied on the spectral measurement of the two green fluorescence bands of the Er ions, followed by data analysis to determine the ratio of the two fluorescence bands as the measure for temperature. This optical read-out method hampers fast temperature measurement per pixel in a scanning microscopy system. While the use of the temperature sensitive material in form of micro-scale coating or nano-scale particles allowed localised temperature sensing, it restricted the temperature measurement to the regions near the temperature sensitive material.

An example of measuring the temperature everywhere across a sample over a micro-scale area involved gluing a micron sized Er-Yb doped fluoride glass particle on an atomic force microscope (AFM) tip, which was scanned across a polysilicon resistor strip on a metal-oxide semiconductor^27^. The green fluorescence ratio was measured by splitting the fluorescence light collected by a microscope objective into two beams, each directed to a photomultiplier tube detector. Two interferential filters centred at 520 and at 550 nm with 10 nm bandwidth were placed in front of the two detectors, respectively, allowing direct measurement of the green fluorescence ratio without spectral measurement. The AFM image taken by scanning the AFM tip with glass micro-particle across the sample was correlated with the temperature determined from the green fluorescence ratio of the Er ions within the glass micro-particle. While this technique allowed measuring temperature with nano-scale spatial resolution, the temperature measurement over the scanning region was very slow since the scan speed had to be very slow with only ten points per second to allow a good thermalization of the glass micro-particle with the sample at each measurement point. Furthermore, the temperature accuracy was only ±5 $^{\circ }$C for a temperature measurement range from room temperature to $\approx$100 $^{\circ }$C

In this paper, the temperature sensitive material of Er-Yb doped sodium-zinc-tellurite glass (hereafter referred to as EYT glass) was used in the form of a substrate for the sample to be measured. This allowed good thermalisation between sample and temperature sensitive material homogeneously over the whole sample area. The sample region of interest was imaged with a scanning confocal microscope (SCM) that was connected to an optical system that measured the green fluorescence ratio by splitting the fluorescence light collected from the SCM microscope and filtering each beam prior to fluorescence intensity detection. This system enabled each pixel of the SCM image to be correlated with the temperature determined from the calibrated green fluorescence ratio. This approach allowed fast temperature measurement over an area of sub-mm spatial dimension (200 $\upmu$m $\times$ 200 $\upmu$m) within less than 1 s and with the spatial resolution ($\approx 1.8$ $\upmu$m diameter per pixel) given by the confocal microscope system. We demonstrate the ability of our sensing approach for real-time monitoring of processes with micro-scale spatial resolution homogeneously over an area much larger than the spatial resolution via tracking the evaporative cooling of a water droplet located on the EYT glass as a proof-of-concept experiment.

## Measurement system and technical detail

The temperature sensing mechanism with the EYT glass involves a ratiometric change in the intensity of the two green fluorescence bands of Er$^{3+}$ ions in the glass at 520–560 nm as a result of a temperature change around the ions, i.e. around the glass. The green fluorescence can be excited using either light at shorter wavelengths (typically at either 476 and 514 nm)^28^ via one-photon absorption or at longer wavelengths in the near infrared region (typically at 980 nm) via two-photon upconversion. The one-photon excitation pathway, used to measure features of proteins and tissues^29,30^ results in auto-fluorescence effects which increases the overall background fluorescence, hence reducing sensitivity. In contrast, upconversion utilises infrared excitation and is able to mitigate such issues with background fluorescence. Although we do not monitor biological tissue here, nevertheless we still use the infrared channel to understand the use of our sensor for future biological applications. Accordingly, we investigate the temperature-dependent fluorescence with 980 nm excitation.

**Figure 1:**
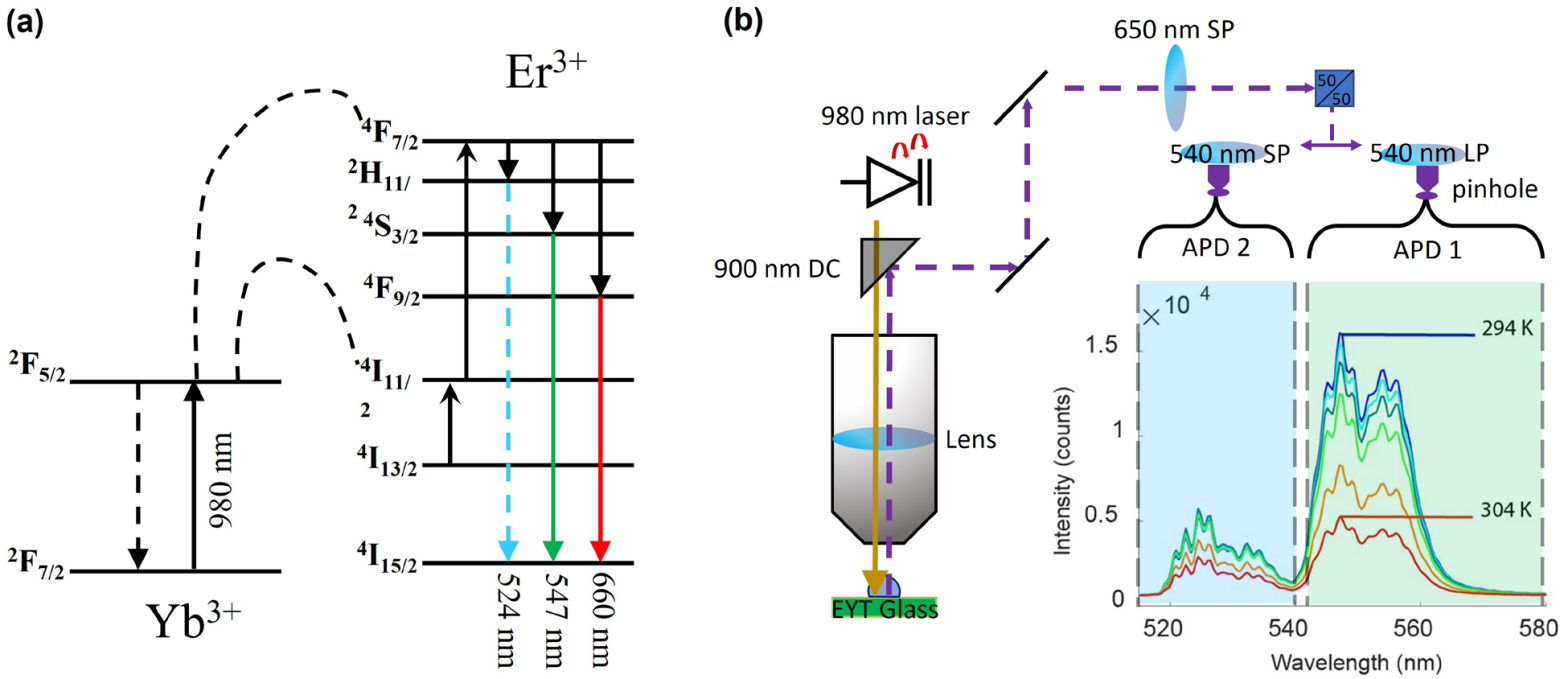
(**a**) The energy level diagram for Er$^{3+}$ and Yb$^{3+}$, where Yb$^{3+}$ acts as a sensitizer in the upconversion energy transfer process to the Er$^{3+}$ $^{4}$I$_{11/2}$ level. The upconversion process upon excitation at 980 nm yields green and red fluorescence of Er$^{3+}$. The two green fluorescence bands at 524 and 547 nm are thermally coupled and hence their intensity ratio is temperature dependent. (**b**) A schematic of the confocal scanning microscopy setup used to measure the green fluorescence ratio with high temporal and spatial resolution. Pump light from the 980 nm laser is filtered via a 900 nm dichroic mirror (DC) where fluorescence from the EYT glass is sent through a series of filters. The total output is first filtered with a 650 nm shortpass filter, and then split, whereby each beam is filtered via a 540 nm shortpass (SP) or 540 nm Longpass (LP) filter, respectively, to measure separately the intensity for each green band with an avalanche photodiode detector (APD).

To significantly increase the Er$^{3+}$ upconversion fluorescence output, we use Yb$^{3+}$, which has been shown to be an effective sensitiser in the upconversion fluorescence process using 980 nm excitation wavelength^21,25^. The 980 nm continuous wave laser pump is absorbed by the Yb$^{3+}$ ions, which transfer the excitation to transitions of the proximal Er$^{3+}$ ions^25^, as shown in Fig. 1a. The two upper levels of the Er$^{3+}$ green fluorescence ($^{2}$H$_{11/2}$ and $^{4}$S$_{3/2}$) are thermally coupled and hence their population ratio *P* is exponentially dependent on the inverse of the temperature via the Boltzmann distribution^31^ described in equation 1, where *A* and *B* are constants; $\Delta E_{f}$ is the energy difference between thermally coupled levels; *k* is the Boltzmann constant and *T* is the temperature of the system.

1 $$\begin{aligned} P = A \exp \ \left( \frac{\Delta E_{f}}{kT}\right) + B \end{aligned}$$

This relation shows that the intensity ratio of the two fluorescence bands of $^{2}$H$_{11/2}$ and $^{4}$S$_{3/2}$ at 524 nm and 547 nm is temperature dependent. Ultimately, this makes the Er$^{3+}$ green fluorescence output a powerful ratiometric sensing approach for temperature.

EYT is a tellurite glass and hence particularly suitable for obtaining Er$^{3+}$ green fluorescence of high intensity for the following reasons. Tellurite glass has a low phonon energy ($\approx 775$ cm$^{-1}$)^32^, resulting in a high quantum efficiency of visible Er$^{3+}$ fluorescence due to low fluorescence quenching. It also has a high rare-earth solubility, allowing for the incorporation of a high concentration of Yb$^{3+}$^33^ to enhance the Er$^{3+}$ fluorescence intensity via efficient upconversion energy transfer. The glass used in this work has the chemical formula of 73 TeO$_{2}$–20 ZnO–5 Na$_{2}$O–2 (Yb,Er)$_{2}$O$_{3}$ with a doping ratio of $1 \times 10^{20}$ cm$^{-3}$ of Er$^{3+}$ and $9 \times 10^{20}$ cm$^{-3}$ of Yb$^{3+}$ and matches the composition of previous work^21^. The EYT glass substrate was fabricated using a previously reported method^34^, where the batch of raw materials was melted in a gold crucible at 830 $^{\circ }$C in open air for approximately 30 min and then cast onto a warm brass plate, which was allowed to cool to room temperature. This resulted in a thin glass piece of approximately $20 \times 15 \times 3$ mm of non-regular dimensions. The top and bottom faces were then mechanically ground and polished to create a flat surface with high optical quality for fluorescence measurement. Some fine scratches were still observed on the surface of the glass. However these did not have a consequential effect on temperature measurements conducted in this work, as the focal spots used for temporal measurements were not collected near to these features.

We measured the ratio of the two bands of the Er$^{3+}$ green fluorescence of the EYT glass confocally via a series of filters, firstly with a 650 nm shortpass filter to remove any contributions related to the $^{4}$F$_{9/2}$ red fluorescence band at  660 nm as any signal from this fluorescence may add some perturbation to the information received by each of our photo-diode detectors. The thus filtered fluorescence signal is then simultaneously split 50/50 into two beams, and then each beam is filtered with a 540 nm shortpass and longpass filter, respectively, making each avalanche photo-diode detector (APD) to record only the green fluorescence bands of the $^{2}$H$_{11/2}$ or the $^{4}$S$_{3/2}$ level, as schematically shown in Fig. 1b. This approach enables fast measurement of the Er$^{3+}$ green fluorescence ratio per confocal pixel and hence fast thermal mapping of the EYT glass surface with micrometer resolution via confocal scanning. For this case the spot size of the confocal beam is defined by the excitation wavelength (980 nm) and the numerical aperture of the objective lens (Model LCPLN50XIR, 0.65 NA, Olympus, Japan). The lateral resolution of the system is given by the size of the excitation beam focused onto the EYT glass, whereby the beam size is determined by the excitation wavelength of the laser and the numerical aperture of the objective lens. The beam size was estimated using the Airy disk diameter approach, which yields a beam size of $\approx 1.8\;\upmu$m for 980 nm excitation wavelength and 0.65 numerical aperture of the objective lens. To mitigate any potential lensing effects introduced by any objects in intimate contact with the glass, we used an objective with a long working distance (14 mm) and adaptive collar correction that is able to take into account the refractive index change between different forms of media.

To achieve consistent measurement of the green fluorescence ratio between each scan, the optimal focal length of the objective lens onto the surface of the glass was found by identifying the z-position of the scanning stage that yielded the strongest signal (highest counts as measured by both of the APDs). This ensured that we only monitored fluorescence from the surface of the glass, thus keeping the focal plane consistent between each scan. We also note that the approximate laser power was roughly 30 $\mu$W at the face of the objective lens which we deem to have minimal localised heating effects as this power regime is too low to observe any large changes in temperature.

## Calibration

To be able to monitor the temperature with the EYT glass and the confocal read-out system, the change in measured fluorescence ratio was calibrated against temperature. For the calibration process, the EYT glass was placed within the vacuum chamber of a cryostat (Cryostation, Montana Instruments, USA) that had a stage with in-built platinum resistor thermometer. The EYT glass was affixed on the cryostate stage with thermal grease to ensure good thermal contact between the glass and the in-built thermometer. The thermal grease also ensured that there was no air-gap between the sample and the cryostat stage. Measurements were then taken from the cryostat stage thermometer.

The fluorescence ratio against temperature was calibrated by varying the temperature of the tellurite glass substrate in the range of 287–307 K via the cryostat and reading the temperature measured by the in-built thermometer on the stage. We made the assumption that the temperature measured within the cryostat chamber is identical with the glass temperature due to good thermal contact. Each measurement was acquired when the temperature stability of the cryostat reached a threshold of $\sim \pm 20$ mK.

**Figure 2:**
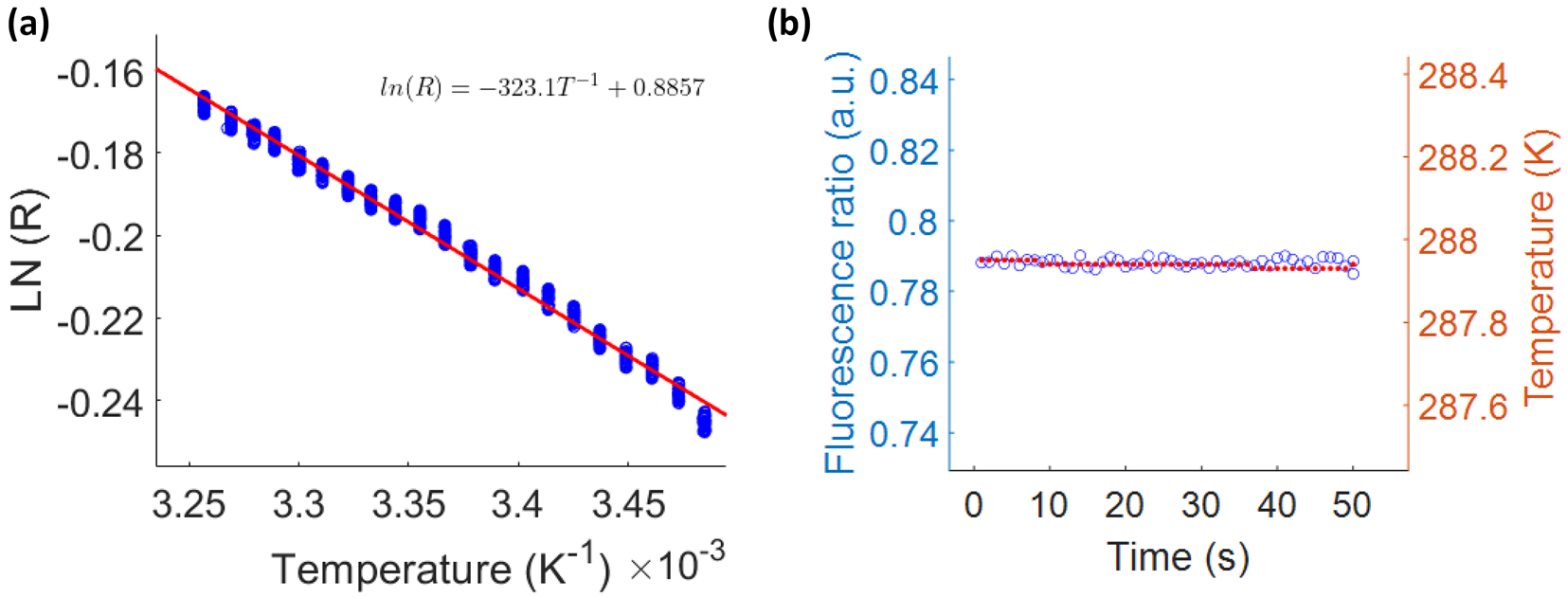
(**a**) The calibration curve of ln(*R*) measured with confocal setup and *1/T* measured with the cryostat thermocouple for a range of set temperatures. For each set temperature, the measurement was conducted over a time period of 50 s. The line represents the linear regression of the measured data points. (**b**) The time trace of the fluorescence ratio measured with the confocal setup (left axis) and corresponding temperature measured with the cryostat sensor (right axis) for 50 s at the set temperature of 288 K. The average standard deviations for the fluctuations of *R* and *T* are $\pm 0.03$ and $\pm 0.03$ K, respectively over a temperature range of 287–307 K.

The population of the $^{2}$H$_{11/2}$ and $^{4}$S$_{3/2}$ levels follows the Boltzmann distribution, thus it is appropriate to express the relationship between fluorescence ratio (*R*) and absolute temperature (*T*) as the natural logarithm of *R* as a function of 1/*T*. Figure 2a shows the log ratio of the green fluorescence ratio as a function of the inverse glass temperature. For each set temperature, the green fluorescence ratio was measured over a 50 s time period. Due to the presence of natural temperature fluctuations of the cryostat chamber, the fluorescence ratio showed some fluctuation, resulting in a spread of the fluorescence ratio for each set temperature (Fig. 2a). An example of the typical thermal stability for a set temperature is shown in Fig. 2b for 288 K over the 50 s measurement time period. In accordance with the Boltzmann distribution of the population of the two green fluorescence levels, a linear trend of ln(*R*) vs 1/*T* is obtained (Fig. 2a). This measured relationship was used to calculate the temperature from the measured fluorescence ratio signal for the proof-of-concept example of an evaporating water droplet on the EYT glass.

In previous work involving ratiometric fluorescence calibration measurements of sodium-zinc-tellurite glass of the same composition as our EYT^21^ or a zinc-tellurite glass with different composition^35^, the change in fluorescence ratio *R* was approximated to be linear with temperature *T*. The sensitivity d*R*/d*T* in these studies was found to be $3.9 \times 10^{-3}$ K$^{-1}$ for the sodium-zinc-tellurite glass in Schartner et al.^21^ and $7.2 \times 10^{-3}$ K$^{-1}$ for the zinc-tellurite glass in Tabanli et al.^35^. To compare the sensitivity of our EYT sensor with that of the previous tellurite glass sensors, we plotted R versus T as shown in Sect. 1 of the Supplementary material, whereby the linear plotting yielded a sensitivity d*R*/d*T* of $3.1 \times 10^{-3}$ K$^{-1}$, which compares well with the previous work on sodium-zinc-tellurite glass^21^ of the same composition as our EYT glass.

## Proof of concept, an evaporating water droplet

**Figure 3:**
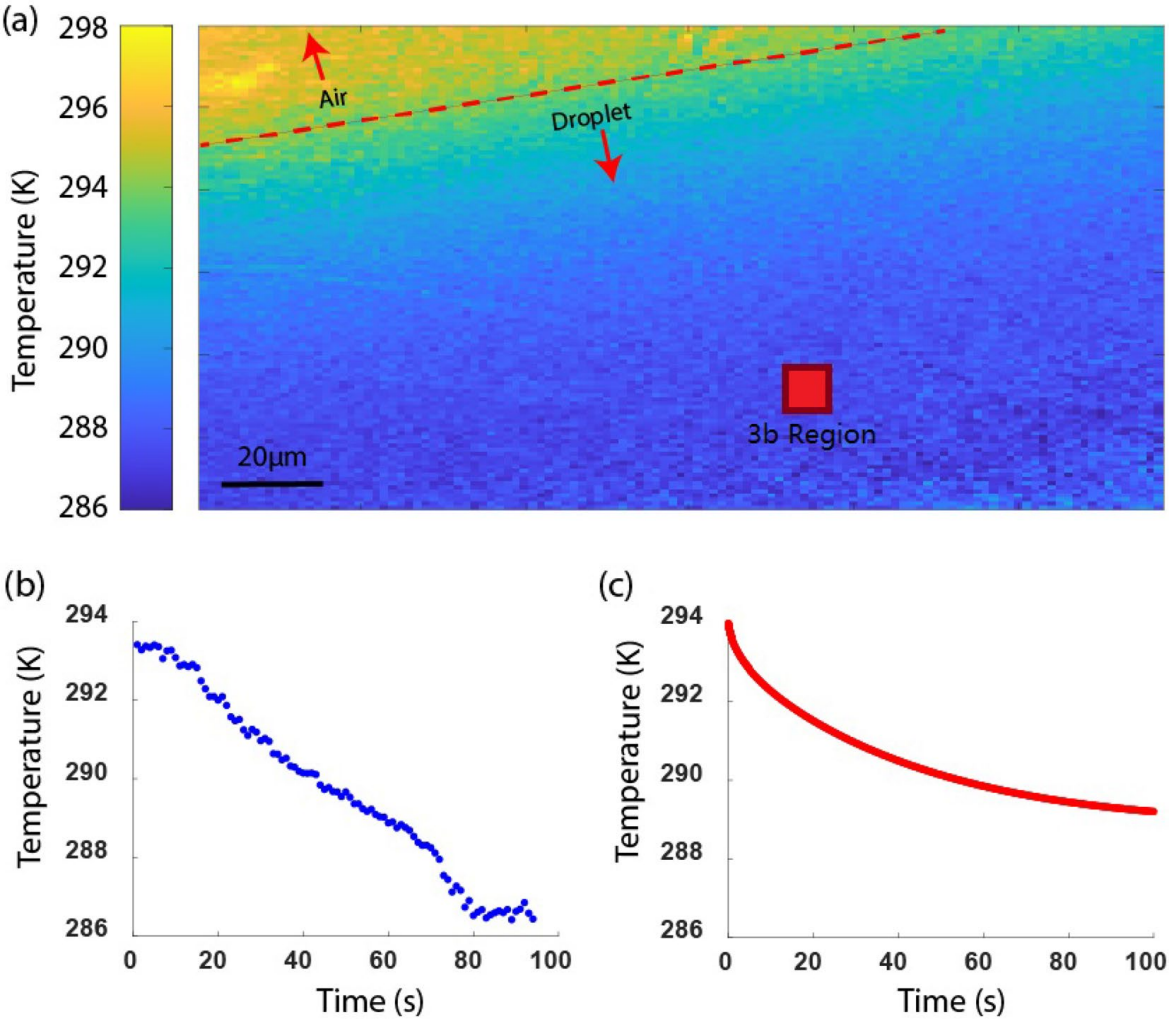
(**a**) Temperature map of the EYT glass with a large area covered by a water droplet. The temperature was obtained from the measured fluorescence ratio *R* and using the calibration curve between ln(*R*) and *1/T*. The cooler area of the glass (blue) corresponds to the droplet, in contrast to the warmer area (orange) occupied by air. The EYT glass covered by air was at 296 K, corresponding to the ambient temperature, whereas the evaporation of the droplet led to a measurable reduction in temperature under the droplet, with minimum recorded temperature of 286 K. (**b**) A decrease in temperature is observed as a function of water droplet evaporation over a time period of 80 s by continuously measuring the fluorescence ratio. (**c**) The expected temperature profile of an evaporating sessile droplet over time, where we assume air convection in the vicinity of the glass plays a significant role on the evaporation rate and hence the temperature decrease of the EYT glass.

The evaporative cooling effect of a water droplet was measured by pipetting 0.5 $\upmu$L of water on the EYT glass. Figure 3a shows the scanning confocal temperature map for the EYT glass with droplet, with 1 $\upmu$m resolution as determined by the step size of the scanning piezo stage. The temperature map shows the EYT glass area that is covered by the droplet (blue) has a lower temperature compared to the EYT glass area without droplet , i.e. the EYT glass area covered by air. The boundary between water and air is clearly seen, indicating discrete change in temperature between the two media. As we do not measure any scattering effects in the confocal scan in Fig. 3a, we assume that the droplet is spread uniformly along the glass surface, as this is also apparent when larger droplets are placed on the EYT and observed by eye.

We measured the temporal temperature change of the droplet as it evaporated by monitoring the average fluorescence ratio of the $10\;\upmu$m $\times$ 10 $\upmu$m region at the marked location (red square) of the EYT glass area covered by the droplet over a time period of 80 s with 1 s measurement intervals (Fig. 3b). The temperature was measured to decrease by 6.97 ± 0.03 K over 80 s. This result agrees with infrared thermography measurement^36^, where a similar temperature decrease of 6 K over a similar time frame was reported for evaporating water droplets of similar size (2 mm in diameter).

Evaporation of droplets of this size has also been studied with emission diffusion computational models^37^ showing that total evaporation can take up to one hour given an ambient temperature of 21 ${^{\circ }\text {C}}$ and humidity of 50%. We note that the droplet size is expected to change due to evaporation as described by Eq. (6) in the Supplementary material, however we note that this process occurs at timescales much larger than the duration of our temporal measurement. For longer timescales, we expect the volume change of the drop to be approximately proportional $t^{3/2}$^38,39^. As our measurement occurred at a much shorter timescale of 80 s, the change in droplet size over the measurement time is expected to be negligible. In Sect. 2 of the Supplementary material, we present a model of evaporation of the water droplet on the EYT glass and use a similar treatment to describe evaporation, and treat thermal diffusion, airflow and mass flux as the dominant forces that drive temperature changes in the evaporation process. To take these parameters into account, we used four times the flux obtained from Eq. (4) in Eq. (5) in the Supplementary information to obtain the temperatures of the droplet and the EYT glass. The modelled decrease of the temperature at the centre of the droplet with evaporation time is shown in Fig. 3c, which shows the temperature at the centre of the droplet decreases during evaporation. The same trend is observed in our experiment as a linear temperature decay, whereas the model displays exponential decay. Conditions such as humidity and temperature were recorded in the laboratory during measurements and were factored into the diffusion model, however this does not directly describe the airflow of the small channel between the objective lens and the EYT glass. The assumption we make is that changes in the droplet mass is the dominant factor of the temperature decrease ignoring any contributions of airflow to evaporation. We note that parameters such as these explain the difference between experiment and model.

## Thermal transport model

**Figure 4:**
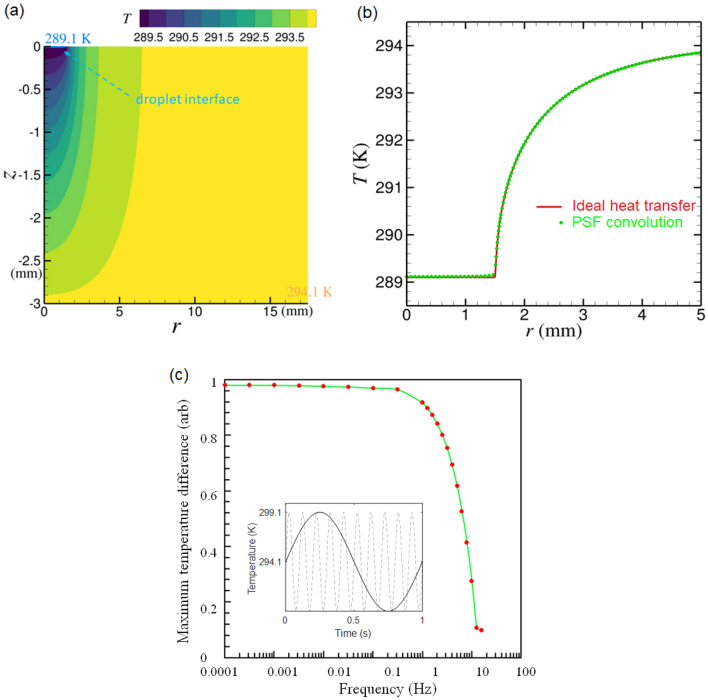
(**a**) A thermal distribution model of EYT glass given a water droplet placed on the top surface with a temperature of 289.1 K. (**b**) The ideal heat transfer model and PSF calculations for the temperature distribution along the top surface of the glass given that all other surfaces are set to an ambient room temperature of 294 K. (**c**) The maximum measurable temperature difference for a harmonic oscillating heat source at varying frequency regimes. The heat source is centred at 294.1 K with an amplitude of 5 K (inset 1 Hz and 10 Hz) where we show measurements above $\approx$1 Hz have the least variance in temperature suggesting the temporal limitations of the system are from 1 Hz and below.

When placing a droplet on the EYT glass slide, the heat transport within the EYT glass medium is anticipated to result in a thermal gradient in depth and lateral direction of the glass slide. This would lead to a gradient in fluorescence ratio when collecting signals from progressively deeper layers in the glass slide and further away from the water droplet acting as heat sink. To understand this effect, we used modelling of the thermal diffusion due to a heat source (sink) that is in intimate contact on the glass surface. We started with the case when the heat source is at a constant temperature of 5 K lower than the ambient temperature of 294.1 K. The heat sink is set as a cylindrical disk with radius of 1 mm (to represent the droplet) which is concentric on top of the cylindrical glass slide of 17.5 mm in radius and 3 mm in thickness, as illustrated in Fig. 4a. We first solved the temperature distribution in the glass medium using the numerical method^40,41^ of a static case, as given in the Supplementary materials, where the glass top surface (in contact with the droplet) transports heat in an adiabatic manner and the other glass surfaces (side and bottom) are fixed at ambient room temperature. The temperature profile, $T_{\text {num}}$, in the glass medium for this static case is shown in Fig. 4a. To mimic the process of temperature sensing using the the EYT glass, we used two point spread functions (PSF), one represents the exciting (absorption) probability distribution, $P_{A}$, in the glass medium and the other, $P_{E}$, represents the fluorescence ratio probability distribution. $P_{A}$ has a Gaussian profile having a waist diameter of 1.8 $\upmu$m and peak wavelength of 980 nm. $P_{E}$ has numerical value between 0 and 1 and is defined as the fluorescence ratio.

The temperature interpreted from the EYT glass fluorescence emission can be estimated as $T_{\text {PSF}} = \sum P_{A} P_{E} T_{\text {num}}$ in which $\sum P_{A} P_{E} = 1$. As shown in Fig.  4b, the temperature measured by the fluorescence from the EYT glass on the glass top surface agrees well with the numerical results from solving the heat transfer equation for the static case.

The second case that we considered is when the temperature of the heat source oscillates with amplitude of 5 K and time harmonic of $\exp {(-i 2\pi f t)}$ where *f* is the oscillation frequency. We followed the same simulation procedure described above for the static case to compare the numerical temperature profile and the temperature measured by the EYT glass from fluorescence emission. The maximum difference of the temperature on the glass top surface as a function of frequency (Fig. 4c) shows that 90$\%$ of the total temperature change can be measured at a temporal resolution of 1 Hz.

## Summary

We show millisecond measurements of temperature, combined with a spatial resolution on the micro scale over a sub millimetre field of view. Simultaneous temporal measurements are provided with an accuracy of $\pm 0.03$ K on a fabricated EYT glass substrate. We showcase our fast micron-scale thermal sensing modality by measuring changes in temperature during evaporation of a droplet of water. The sensing platform was able to build up a temperature map that is able to distinguish the thermal gradient within the droplet. Over a total time period of 80 s, with measurements acquired at 1 s intervals, we measured a temperature decrease of 6.97 K due to the cooling effect of a water droplet. This result is also in agreement with our mathematical model, that assumes mass flux as the main observable component behind evaporation of sessile water droplet. In future applications, we anticipate that our temperature sensing approach may have value in measuring small scale temperature changes in fundamental processes in biological and chemical systems.

### Supplementary Information

Supplementary Information.

## Data Availability

All data generated or analysed during this study are included in this published article and its nnnn information files. Any RAW datasets can be made available from the corresponding author upon reasonable request.
