# Supplementary material for: Continuous spatial field confocal thermometry using lanthanide doped tellurite glass
Source: Sci Rep. 2024 Jul 2;14:15084. doi: 10.1038/s41598-024-65529-9 (PMC11219951; doi:10.1038/s41598-024-65529-9)
Supplement: Supplementary file 1 — Supplementary Information. [file 41598_2024_65529_MOESM1_ESM.pdf]

Supplementary Material: Continuous spatial field confocal thermometry using lanthanide doped tellurite glass

daniel.stavrevski@student.rmit.edu.au

1 EYT glass calibration measurement

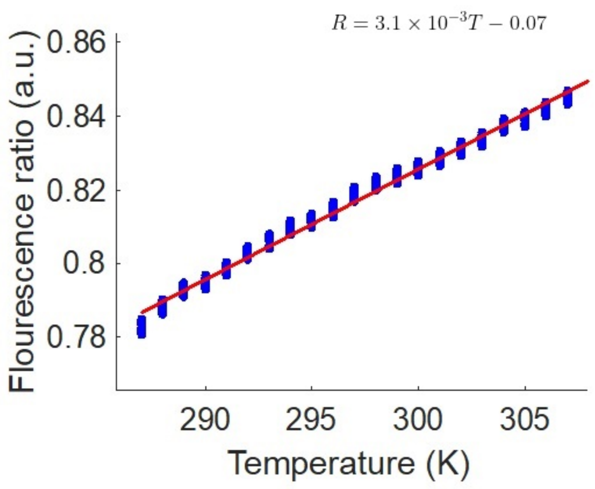

Supplementary Figure 1. A calibration curve of fluorescence ratio R measured with confocal setup and temperature T measured with the cryostat thermocouple for a range of set temperatures. Each set temperature was conducted over 50 seconds, where the line of best fit is used to determine the sensitivity of the EYT glass.

2 Mathematical model for evaporation of a sessile water droplet on newly developed tellurite glass thermometer

We demonstrate a mathematical model to study the evaporation of the axis-symmetric sessile water droplet (with respect to the beam axis), and relate this to the estimates of temperature acquired using the technique presented in this paper. The droplet with constant density $\rho^w = 9.98 \times 10^{-4}$ g/mm^3, specific heat $c_p^w = 4.18 \times 10^9$ mm^2/s$^2 \cdot$ K and thermal conductivity $k^w = 6.04 \times 10^5$ g $\cdot$ mm/s$^3 \cdot$ K[1] sitting on a horizontal substrate of constant density $\rho^s = 5.2 \times 10^{-2}$ g/mm^3, specific heat $c_p^s = 2.91 \times 10^8$ mm^2/s$^2 \cdot$ K and thermal conductivity $k^s = 1.15 \times 10^6$ g $\cdot$ mm/s$^3 \cdot$ K[2]. As shown in Fig. 2, a cylindrical coordinate system (r, ϕ, z) is employed with an origin located at the centre of the droplet on the substrate with its z-axis pointing opposite to the beam direction. As such, the upper surface of the substrate is then at $z = 0$, the lower side is at $z = -h^s$ with $h^s = 3$ mm thickness of the substrate, and its radial size is $R^s = 17.5$ mm. Also, the shape of the free surface of the droplet at time t is described as $z = h^s(r,t)$, and the constant atmospheric temperature of the surrounding atmosphere is $T^a = 294.1$ K.

The water droplet is taken as an incompressible Newtonian fluid, and the inertial and thermal convection effects are negligible[3]. Also, we assume that the surface-tension dominates the gravitational effects, so that the profile of the free surface of the droplet can be well approximated by a spherical cap as:

$$h(r,t) = \sqrt{\left(\frac{R^w}{\sin\theta}\right)^2 - r^2} - \frac{R^w}{\tan\theta} \tag{1}$$

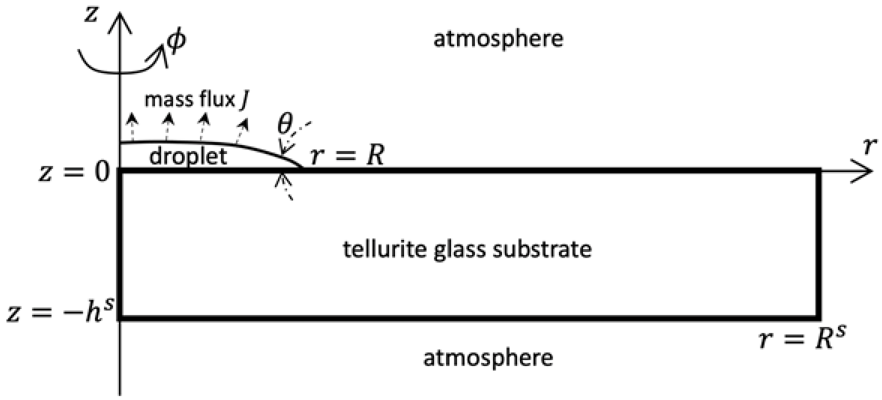

Supplementary Figure 2. Sketch of evaporation of a sessile water droplet on a horizontal tellurite glass substrate. The tellurite glass substrate is in the shape of a circular disk which radius is R^s and height is h^s. The free surface of the droplet is approximated by a spherical cap with contact angle θ and contact line radius R^w. The evaporation process is illustrated by the diffusion of water mass flux J from the droplet free surface to the surrounding atmosphere. A cylindrical coordinate system (r, ϕ, z) is set with an origin located at the centre of the droplet on the substrate with its z-axis pointing opposite to the beam direction and ϕ representing the symmetry with regard to the right-hand rule.

where $\theta \equiv \theta(t)$ is the contact angle, and R^w is the drop radius on the substrate. Since the glass substrate used in our experiments is hydrophobic, and the evaporation rate of the droplet is much slow relative to its lifetime, we can assume that R^w is constant, and the diffusion of the vapour concentration from the droplet to the surrounding is quasi-steady. As a result, the atmosphere vapour concentration $c^a \equiv c(z,r,t)$ satisfies the Laplace equation:

$$\nabla^2 c^a = 0. \tag{2}$$

We assume that the vapour concentration is saturated in the layer of the atmosphere just above the free surface of the droplet, which leads to $c^a = c^a_{sat}(T^a) = 1.88 \times 10^-8$ g/mm^3 on $z = h(r,t)$ and $r < R^w$[4]. Also, on the unwetted surfaces of the substrate, there is no mass flux as $\nabla c^a \cdot n = 0$ where n is the unit normal vector pointing into the substrate. Far from the droplet and substrate, the concentration of vapour goes to the ambient value of the room as $c^a = H^a c^a_{sat}(T^a)$ in which $H^a = 0$ is the room relative humidity. Eq. (2) can be efficiently solved by the non-singular boundary element method[5,6]. Once c^a is obtained, the local mass flux $J(r,t)$ from the droplet to the surrounding atmosphere can evaluated by:

$$J(r,t) = D^a \nabla c^a \cdot n. \tag{3}$$

in which $D^a = 24.4$ mm^2/s[3] is the vapour diffusion coefficient in air.

To balance this mass flux, local energy is needed from the droplet:

$$\mathscr{L}_h J(r,t) = k^w \nabla T^w \cdot n \tag{4}$$

on the free surface $z = h(r,t)$ and $r < R^w$ where $\mathscr{L}_h \equiv \mathscr{L}_h(T^a) = 2.45 \times 10^{12}$ mm^2/s^2[1] is the latent heat, and $\nabla T^w \cdot n$ is normal gradient of the drop temperature on the free surface. In our experiments, since the effect of thermal convection is much smaller relative to the thermal diffusion, the droplet and substrate temperatures satisfy:

$$\frac{\partial T^w}{\partial t} - \frac{k^w}{\rho^w c^w_p} \nabla^2 T^w = 0, \tag{5a}$$

$$\frac{\partial T^s}{\partial t} - \frac{k^s}{\rho^s c^s_p} \nabla^2 T^s = 0, \tag{5b}$$

where $T^w \equiv T^w(r,z,t)$ and $T^s \equiv T^s(r,z,t)$ are the droplet and substrate temperatures, respectively. On the wetted surface of the substrate (the interface between the droplet and substrate) where $z = 0$ and $r < R^w$, both the temperature and heat flux should be continuous,

$$T^w = T^s, \tag{6a}$$

$$-k^w \frac{\partial T^w}{\partial z} = -k^s \frac{\partial T^s}{\partial z}. \tag{6b}$$

For the rest surfaces of the substrate, we let $T^s = T^a$. Eq (5a) can be solved by using the Crank–Nicolson method[7].

The evaporation of a droplet is a dynamic procedure that can be tracked by its volume change:

$$\frac{\mathrm{d}V}{\mathrm{d}t} = \frac{2\pi}{\rho^w} \int_0^{R^w} J \sqrt{1 + \left(\frac{\partial h}{\partial r}\right)^2}\, r\, \mathrm{d}r \tag{7}$$

where V is the droplet volume as

$$V = \frac{\pi h_m [3(R^w)^2 + h_m^2]}{6} \tag{8}$$

with

$$h_m = R^w \tan\left(\frac{\theta}{2}\right). \tag{9}$$

In our experiment, at the initial stage $t = 0$, a droplet with volume $V = 0.5\ \mu$L is injected on the substrate which radius $R^w = 1.5$ mm. From Eqs. (8) and (9), the contact angle θ is obtained, and then the free surface shape of the droplet is evaluated by using Eq. (9). By solving Eq. (2), we can calculate the local mass flux J in Eq. (3) which is introduced to Eq. (4) as the boundary condition for Eq. (5a) to compute the temperature profiles. From the local mass flux J, we can also compute the volume change of the droplet by using Eq. (7), and then calculate the new volume of the droplet by using the Euler time advancing scheme. The calculations are iterated to compute the temperature measured at multiple time points as the droplet evaporates.

References

1. Lide, D. R. & Kehiaian, H. V. *CRC Handbook of Thermophysical and Thermochemical Data* (CRC Press, 2020).
2. Kutin, A., Plekhovich, A., Balueva, K. V., Motorin, S. & Dorofeev, V. Thermal properties of high purity zinc-tellurite glasses for fiber-optics. *Thermochimica Acta* **673**, 192–197, DOI: 10.1016/j.tca.2019.01.027 (2019).
3. Dunn, G. J., Wilson, S. K., Duffy, B. R., David, S. & Sefiane, K. The strong influence of substrate conductivity on droplet evaporation. *J. Fluid Mech.* **623**, 329–351, DOI: 10.1017/s0022112008005004 (2009).
4. Popov, Y. O. Evaporative deposition patterns: Spatial dimensions of the deposit. *Phys. Rev. E* **71**, DOI: 10.1103/physreve.71.036313 (2005).
5. Klaseboer, E., Sun, Q. & Chan, D. Y. C. Non-singular boundary integral methods for fluid mechanics applications. *J. Fluid Mech.* **696**, 468–478, DOI: 10.1017/jfm.2012.71 (2012).
6. Sun, Q., Klaseboer, E., Khoo, B. C. & Chan, D. Y. A robust and non-singular formulation of the boundary integral method for the potential problem. *Eng. Analysis with Boundary Elem.* **43**, 117–123, DOI: 10.1016/j.enganabound.2014.03.010 (2014).
7. Crank, J. & Nicolson, P. A practical method for numerical evaluation of solutions of partial differential equations of the heat-conduction type. *Math. Proc. Camb. Philos. Soc.* **43**, 50–67, DOI: 10.1017/s0305004100023197 (1947).
